# Role of nano-hydrogels coated exosomes in bone tissue repair

**DOI:** 10.3389/fbioe.2023.1167012

**Published:** 2023-05-09

**Authors:** Yuqi Pan, Yige Li, Wenjun Dong, Bowei Jiang, Yuhao Yu, Yunsu Chen

**Affiliations:** ^1^ Department of Joint Surgery, Shanghai Sixth People’s Hospital Affiliated to Shanghai Jiao Tong University School of Medicine, Shanghai, China; ^2^ Department of Rehabilitation, The Second Affiliated Hospital of Nanjing Medical University, Nanjing, China

**Keywords:** hydrogels, exosomes, hydrogels coated exosomes, bone tissue, repair and regeneration

## Abstract

With the development of nanotechnology, nanomaterials are widely applied in different areas. Some nanomaterials are designed to be biocompatible and can be used in the medical field, playing an important role in disease treatment. Exosomes are nanoscale vesicles with a diameter of 30–200 nm. Studies have shown that exosomes have the effect of angiogenesis, tissue (skin, tendon, cartilage, et al.) repair and reconstruction. Nano-hydrogels are hydrogels with a diameter of 200 nm or less and can be used as the carrier to transport the exosomes into the body. Some orthopedic diseases, such as bone defects and bone infections, are difficult to handle. The emergence of nano-hydrogels coated exosomes may provide a new idea to solve these problems, improving the prognosis of patients. This review summarizes the function of nano-hydrogels coated exosomes in bone tissue repair, intending to illustrate the potential use and application of nano-hydrogels coated exosomes in bone disease.

## Introduction

Bone tissue can regenerate as part of the repair process following bone disease, such as trauma, bone tumor, and bone defect. The commonest form of bone repair is bone fracture healing. Intramembranous and endochondral ossification are involved in the process (Dimitriou et al., 2011). However, the ability of bone tissue repair is limited. In severe bone diseases, the bone’s compensatory capacity is impaired, in which interventions are needed. Sometimes it is necessary to perform surgery because conservative therapies usually have little effect on bone tissue repair. Although surgery can achieve good results in most cases, it is, after all, an invasive procedure, and in patients who already had bone surgery before, a second revision surgery requires careful consideration. So, finding ways to reduce or even avoid surgery intervention and effectively promote bone repair is a new trend in the treatment of bone diseases.

Johnstone et al. discovered a vesicle-like structure during the period of sheep reticulocyte maturation and isolated these membrane-bound vesicles by ultracentrifugation. The vesicle-like structures were given the name “exosomes” for the first time (Johnstone et al., 1987; Johnstone, 2005). Exosomes are nanoscale extracellular vesicles with 30–200 nm in diameter that are produced through budding from the plasma membrane and endosome membrane. Exosome contain a variety of substances, such as nucleic acids, lipids, and proteins, expressing transmembrane proteins and receptors that facilitate intracellular communication and materials transportation. Donor cells can transfer exogenous substances such as proteins, mRNAs, microRNAs (miRNAs), and lipids to recipient cells via exosomes (Liang et al., 2021a). Exosomes carry mRNAs and miRNAs, playing a key role in the repair of various tissue injuries (Wang et al., 2020a; Liu et al., 2021). It has been shown that mesenchymal stem cells (MSCs) derived exosomes can protect cartilage, downregulate the expression of inflammatory factors, and inhibit the conversion of macrophages to the M1 type (M1 type promotes inflammatory reaction), which can be used as a novel treatment for osteoarthritis (Pandey et al., 2022). Bone morphogenetic protein 2 (BMP2)/macrophage-derived exosomes can regulate the osteoblast differentiation of MSCs, stimulating bone regeneration. Exosomes also play an important role in angiogenesis and in the repair and reconstruction process of many tissues (e.g., skin, tendon, cartilage) (Zhang et al., 2021a). The excellent properties of exosomes make them ideal materials for bone tissue repair. Many studies have focused on this hot spot.

On the other hand, cutting-edge research in nanomaterials also brings new opportunities for bone repair and reconstruction. Hydrogels are a class of polymeric materials with high water contents that have been widely used in cell culture, drug delivery, tissue engineering, and other biomedical fields. Hydrogels have a unique three-dimensional structure that provides sufficient space to accommodate a variety of substances, including small molecules, polymers, and particles. Due to their soft, moist, and biocompatible properties, hydrogels can be used as matrix components for engineering living cells, leading the nascent field of engineering material science (Liu et al., 2022a). Hydrogels can also be used as drug carriers. After relevant modifications, Hydrogels enable precise target positioning and controlled release of drugs, which is safe and effective. Some wearable tissue-adhesive electronic devices also form interfaces with tissues (e.g., skin) through the good adhesion and electrical conductivity of hydrogels, and are used for real-time *in vitro* sensing and organ repair (Li et al., 2021a). The use of biomaterials with antimicrobial hydrogel coatings is an effective way to combat colonizing bacteria. As for bone tissue repair, some studies demonstrated hydrogels can accelerate bone growth (Anada et al., 2019; Hasani-Sadrabadi et al., 2020; Datta et al., 2021), which can be used in the treatment of bone diseases.

According to the previous study, exosomes and hydrogels have positive functions in the process of bone tissue repair, bringing new hope for the treatment of orthopedic diseases. And there are studies focusing on the nano-hydrogels coated exosomes in bone tissue repair aspects.

This review intends to explore the role of nano-hydrogels coated exosomes in the bone tissue repair process and discover its functions in various bone diseases.

## Exosomes

**FIGURE 1:**
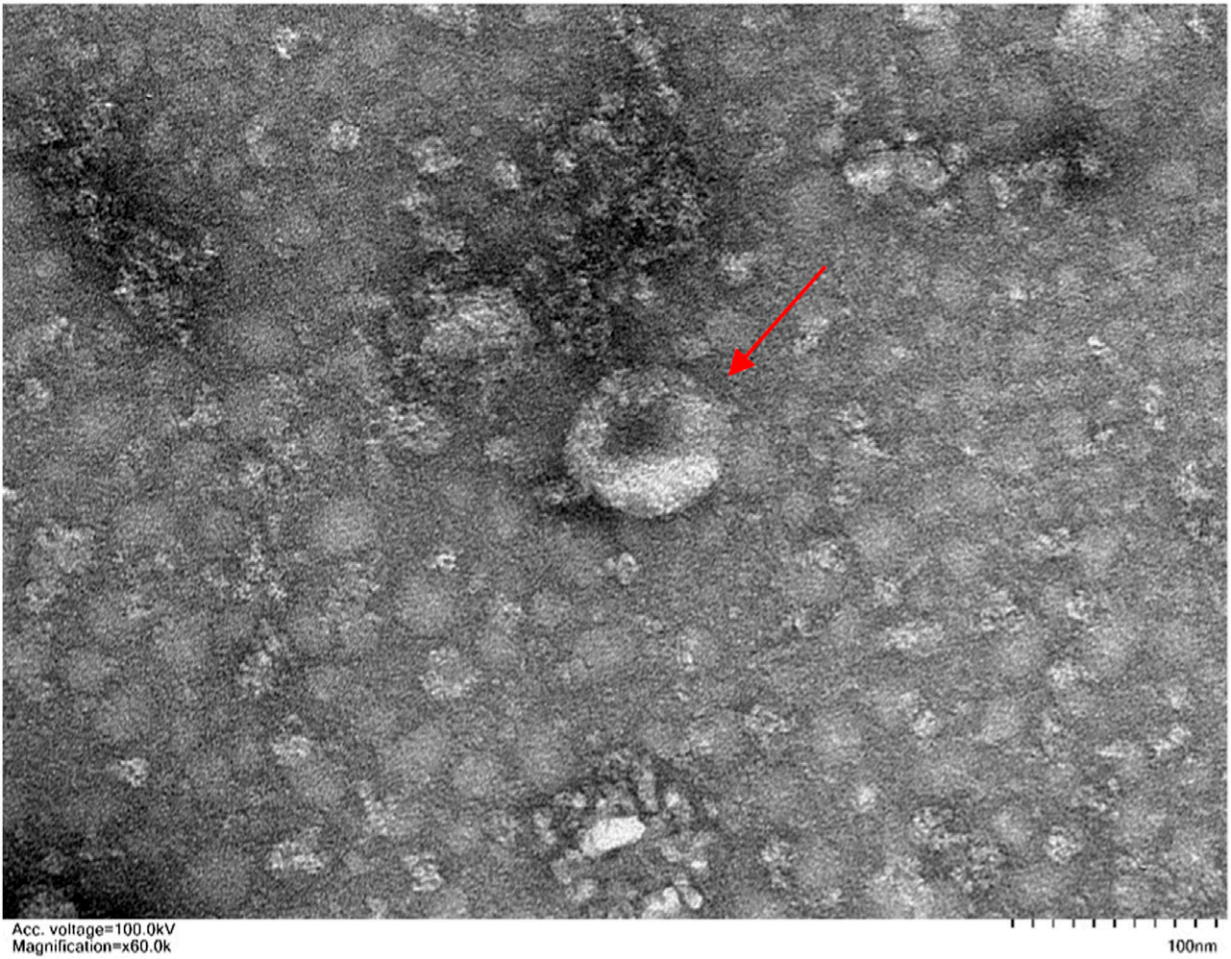
Microscopic observation of exosomes (magnification × 60 k, Scale 100 nm).

**FIGURE 2:**
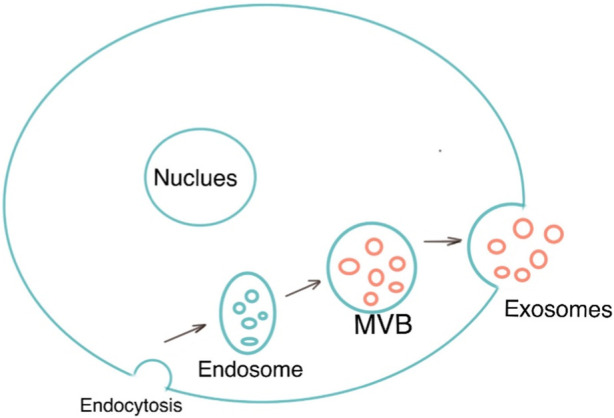
The process of exosomes secretion in cells.

Exosomes are single-membrane vesicles with the same topology as cells, with diameters ranging from 30 to 200 nm (Pegtel and Gould, 2019). Exosomes are a subset of extracellular vesicles which are produced as a result of double invagination of the plasma membrane and the formation of intracellular multivesicular bodies (MVBs) containing intraluminal vesicles (ILVs). ILVs are eventually secreted as exosomes with diameters ranging from 40 to 160 nm via fusions of MVBs to the plasma membrane and exocytosis (Kalluri and LeBleu, 2020). MVBs and exosomes’ production and release are controlled by the endosomal sorting complexes needed for the transport (ESCRT) pathway (Wollert and Hurley, 2010). Exosomes are now known to be released into the extracellular environment by donor cells to perform a variety of biological functions, such as intracellular communication and the exchange of genetic material and proteins between a parent cell and surrounding cells. Their functions for drug delivery in cancer immunotherapy have been proven. Because of their microRNA and mRNA contents, they are also a promising biological gene delivery system (Hade et al., 2021). As a novel material, its application in the medical field remains to be explored further. Figure 1 shows the morphology of exosomes (Red arrow) under electron microscopy. Figure 2 is the diagram of exosomes secretion.

### Exosomes components

Exosomes are made up of a wide range of materials, including lipids, proteins, DNA, and RNA. Some specific components, including lipids, proteins, DNA, mRNA, and noncoding RNAs, can function as autocrine and/or paracrine agents (Dai et al., 2020).

#### Lipid

Exosomes’ lipid composition includes sphingolipids, cholesterol, phosphatidylserine, saturated fatty acids, and ceramides, all of which can be discovered in plasma membranes (Skotland et al., 2020). Exosomes’ lipid composition is cell-specific or conserved. Lipids have a crucial role in exosomes shape preservation, exosomes synthesis, and exosomes’ homeostasis maintenance in the recipient cells (Mashouri et al., 2019).

#### Protein

Exosomes’ protein composition includes membrane trafficking-related proteins, such as the tetraspanins (CD63, CD81, CD82, and CD9) (Larios et al., 2020). Additionally, integrins (cell adhesion-related proteins), actin, myosin (participating in cytoskeletal construction), MHC class II proteins, and heat-shock proteins (Hsp60, Hsp70, and Hsp90) can be concentrated in exosomes (Clayton et al., 2005; Dai et al., 2020). Regardless of the type of cells that exosomes originate from, ESCRT proteins and their accessory proteins (Alix, TSG101, HSC70, and HSP90) are predicted to be present in exosomes as they govern exosomal production and MVBs transit (Tauro et al., 2012). This group of proteins are hence known as “exosomal marker proteins”. (Tauro et al., 2012).

#### DNA and RNA

Exosomal DNA (exoDNA) can be single-stranded or double-stranded DNA. It contains both nuclear and mitochondrial DNA. ExoDNA exists on the surface of the vesicle or inside the vesicle (Wortzel et al., 2019).

The most frequently researched exosomal RNA species are mRNAs and miRNAs (Carnino et al., 2020). Some exosomal mRNAs have been shown by Valadi et al. to be intact and capable of being translated into useful proteins in recipient cells (Valadi et al., 2007). MicroRNAs (miRNAs), which are significant members of the small non-coding RNA family and range in length from 20 to 22 nucleotides, have been extensively studied in a wide range of physiological and pathological processes. They mediate post-transcriptional gene silencing by binding to the 3′-untranslated region or open reading frames of the target mRNA (Treiber et al., 2019). MiRNAs can also be found in exosomes that have been extracted from bodily fluids (such as saliva, blood, or serum), suggesting the potential benefits of employing exosomal miRNAs as the novel, non-invasive biomarkers (Nedaeinia et al., 2017). RNA species are sorted into exosomes in a variety of ways.

### Classifications

Depending on whether they have undergone artificial modification, exosomes are classified as natural exosomes or designed exosomes. Natural exosomes can be divided into animal-derived exosomes and plant-derived exosomes. Animal-derived exosomes are further separated into normal exosomes and tumor exosomes according to their environments (normal or malignant) (Zhang et al., 2020a).

### Exosomes from different origins

Nearly all normal cell types, including human umbilical vein endothelial cells, mesenchymal stem cells (MSCs), T cells, B cells, macrophages, dendritic cells (DC), and natural killer (NK) cells, have the ability to create exosomes (Cheng et al., 2017; He et al., 2020; Li et al., 2020; Zhao et al., 2020).

Additionally, the previously described normal exosomes can be found in large quantities in biofluids like saliva, plasma, urine, ascites, milk, and bile.

**FIGURE 3:**
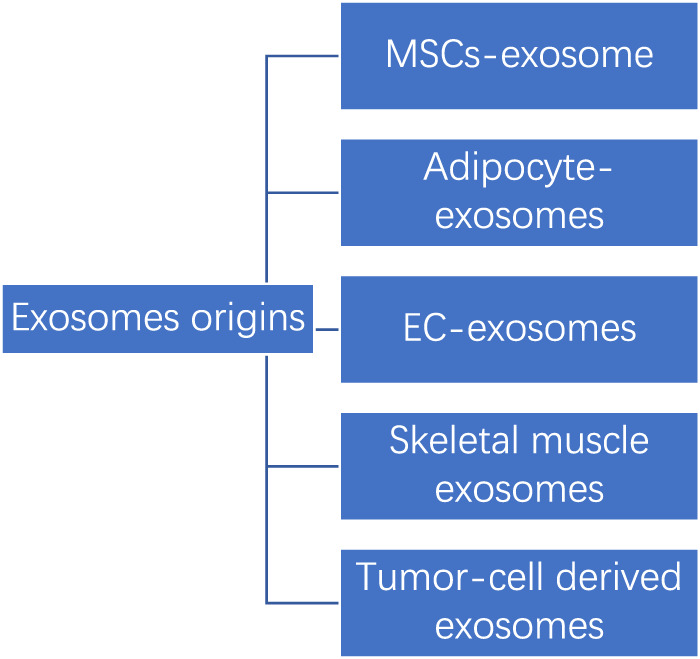
Common exosomes origins.

Below we have listed several common sources of exosomes. (See Figure 3).

#### Mesenchymal stem cells exosomes

Mesenchymal stem cells (MSCs) are multipotent mesenchymal stromal cells that can differentiate into a variety of cell types, including osteocytes, chondrocytes, adipocytes, cardiomyocytes, and endothelial cells. They also have the self-renew ablity (generate more MSCs themselves) (Akbari et al., 2020). MSCs can be extracted from a variety of body fluids and tissues, including adipose tissue, bone marrow, tooth pulp, synovial fluid, amniotic fluid, placenta, umbilical cord, and Wharton’s jelly (Andrzejewska et al., 2019). MSCs exosomes are widely used in tissue repair aspects.

#### Adipocyte exosomes

Adipocytes can secret exosomes and act locally in paracrine ways or enter the bloodstream to exert systemic effects (Isaac et al., 2021). Using a fat-specific Dicer deletion, Thomou et al. demonstrated that adipocytes were a significant source of circulating exosomal miRNAs (Thomou et al., 2017). MiR-16, miR-27a, miR-146b, and miR-222 are found in exosomes generated by giant adipocytes, and they can be transferred to tiny adipocytes to promote lipogenesis and adipocyte hypertrophy (Müller et al., 2011). Adipocyte exosomes also contain non-miRNA that have distant biologic effects (Isaac et al., 2021).

#### Endothelial cell exosomes

The endothelial cell (EC) activation or apoptosis can trigger the release of generated exosomes. In peripheral circulation, EC exosomes account for 5%–15% of the total circulating vesicles (Arraud et al., 2014). Proteins detected in released EC exosomes match those in the generating EC, according to proteomic investigations; some of these proteins can be transported to recipient cells (Liu et al., 2013).

#### Skeletal muscle exosomes

Exosomes produced by skeletal muscle have been found to have both paracrine and endocrine impacts on the preservation of muscle homeostasis and communication with other tissues (Qin and Dallas, 2019). Myoblasts and myotubes, two types of muscle cells, are sources of exosomes that express the Tsg101 and Alix protein markers as well as other signals transduction related proteins (Rome et al., 2019). Exosomes may play a role in the differentiation and maturation of skeletal muscle as numerous proteins involved in the transition from myoblast to myotube are found in exosomes, (Le Bihan et al., 2012).

#### Tumor-cell derived exosomes

Exosomes can be produced in huge quantities by tumor cells. The unique antigens on their surface may reveal information about the origins of donor cells. Tumor exosomes contribute to the growth, metastasis, and immunological control of tumors. They also monitor the onset of diseases and act as diagnostic indicators for illnesses (Poggio et al., 2019; Sanderson et al., 2019).

### Exosomes and bone tissue repair

Exosomes can be secreted by cells such as osteoblasts, osteoclasts, osteocytes, and MSCs, which are known to mediate cellular communication and participate in the regulation of the bone microenvironment. Exosomes play critical regulatory roles in bone remodeling. Exosomes derived from osteoblasts stimulate osteoclast differentiation *in vivo*, and thus exosomes treatment can be used to enhance the removal of damaged tissue (Hade et al., 2021). Exosomes from different origins can be made in different forms and injected into the bone defect area to facilitate bone regeneration. Stem cells derived exosomes can promote osteogenesis through four main mechanisms: reducing apoptosis, recruiting mesenchymal stem cells and promoting their proliferation, creating an osteogenic-inducing environment to promote osteogenic differentiation of stem cells, and accelerating angiogenesis and bone vascularization (Girón et al., 2022). Its applications for bone defect, bone and cartilage regeneration, osteoarthritis, osteoporosis, and osteonecrosis have been described (Bei et al., 2021). The most commonly used exosomes in medical fields are MSCs exosomes. MSCs can secret exosomes, which are the primary therapeutic agents for encouraging tissue regeneration (Yu et al., 2014). MSCs-exosomes have anti‐inflammatory and immunomodulatory functions and can be a perfect alternative to MSCs therapy because they possess similar biological functions to their originating cells while they are stabler and have lower immunogenicity (Ha et al., 2020). MSCs-exosomes were proven to have a protective function against the cardiovascular system by Lai et al. in a cardiac ischemia-reperfusion damage mouse model (Lai et al., 2010). And later, exosomes’ therapeutic use was extended to models of additional diseases. Studies have shown exosomes from MSCs have a significant impact on bone regeneration and exhibit osteoinductive properties (Li et al., 2018).

## Hydrogels

The chemical structure of hydrogels is a three-dimensional polymeric network connecting hydrophilic components (Gradinaru et al., 2018). Hydrogels are made up of hydrophilic polymers with polar functional groups and have a high water content (Zhang et al., 2020b). The network is composed of crosslinking polymers with covalent bonds or noncovalent interactions. Their structure can be changed for different applications. Due to their stable physicochemical properties, hydrogels have been widely studied. The biomedical applications of hydrogels include 3D cell culture, drug delivery, wound dressing, tissue engineering and so on (Ho et al., 2022).

### Common materials of the hydrogels

#### Natural polymers

Natural hydrogels have good biocompatibility and bioactivity, which can promote cell adhesion, proliferation, and tissue repair and regeneration. Natural polymer molecules from living beings, such as proteins and polysaccharides (hyaluronic acid, sodium alginate, chitosan, and agarose), make up the raw ingredients [e.g., collagen, tropocollagen, gelatin, silk fibroin (Ju et al., 2023)]. But they are unstable, needing to be crosslinked with additional polymers due to their poor durability, mechanical characteristics, and tissue adhesion capabilities.

##### Polysaccharides

Polysaccharides that have been used in hydrogels making include hyaluronic acid (HA), chitosan, and alginate.

HA is a linear nonsulfated glycosaminoglycan, which is a significant element of the ECM and is present in practically all bodily fluids and tissues (Yasin et al., 2022). Since HA is an important part of cartilage tissue and has good biocompatibility, HA hydrogels are crucial in the cartilage tissue engineering (Ngadimin et al., 2021).

Chitosan is a frequently used ingredient in the process of creating natural hydrogels, which is hydrophilic, biocompatible, and biodegradable. It can be degraded by lysozyme, acid, and colonic bacteria in the human body (Ways et al., 2018). Chitosan has an amine group and can react with other aldehyde-containing polysaccharides through Schiff base reaction, making it a good material for the preparation of self-healing hydrogels (Mo et al., 2021).

Alginate is a natural marine polysaccharide, and sodium alginate is the most often used extract (Zhang et al., 2021b). Alginate is an abundant and easily accessible biopolymer with exceptional biocompatibility, good porosity, high capacity to retain water, and changeable viscosity (Liu et al., 2022b). Through ion-exchange interactions with cations, sodium alginate has exceptional pH sensitivity and can quickly form gels under incredibly mild conditions (Maity and Das, 2021).

##### Proteins

Proteins used for hydrogels synthesis include collagen, gelatin, silk fibroin (SF), and polydopamine (PDA).

As the most prevalent protein in the ECM, collagen has a triple helix shape that offers high tensile strength. Since natural ECM contains a lot of collagen, collagen-based hydrogels are becoming more and more common as scaffolds for tissue engineering. Type I collagen is by far the most common kind of collagen among the other types. Collagen’s structural flexibility enables cross-linking to create a three-dimensional porous fibrous meshwork that makes extracellular vesicles (Evs) loading easier (Antoine et al., 2014).

The processing, molecular weight, and isoelectric point of collagen have a significant impact on the characteristics of gelatin, a partially hydrolyzed derivative of collagen (Salahuddin et al., 2021). Gelatin, as opposed to collagen, has greater temperature stability and biocompatibility (Mahmood et al., 2022). Gelatin methacrylate (GelMA), a composite improved form of gelatin hydrogel, was created in 2000 by Bulcke et al. (Van Den Bulcke et al., 2000) and is a typical form of hydrogels. Methacrylic anhydride and gelatin make up the photosensitive biohydrogel material known as GelMA, which can be activated by ultraviolet (UV) or visible light to create three-dimensional structures strong enough to sustain cell growth and differentiation (Kurian et al., 2022).

Sericin protein (SF) is a natural polymeric protein polymer extracted from natural silk and widely used in biomanufacturing. SF is highly biocompatible, biodegradable, and has a high tensile biomechanical strength (Lujerdean et al., 2022). Because SF biopolymers naturally form regular-sheet stacks, they can be treated to create hydrogels that are only physically cross-linked without the use of chemical cross-linking agents (Ju et al., 2023).

Polydopamine (PDA) is a biopolymer created when dopamine undergoes oxidative polymerization (Li et al., 2021b). PDA is easily and cheaply made without the use of hazardous solvents, and as a result, it has low cytotoxicity and high biocompatibility (>80%). PDA has also been demonstrated to improve cell adhesion and proliferation (Li et al., 2021b). Additionally, PDA is especially suited for biological applications because of its hydrophilicity and capacity to functionalize a variety of substrates.

#### Synthetic polymers

By crosslinking synthetic hydrophilic polymers, such as polyethylene glycol (PEG), polyvinyl alcohol (PVA), polyacrylic acid and its derivatives, polylactic acid-hydroxy acetic acid copolymer (PLGA), and phenoxyethyl methacrylate (PHEMA), synthetic hydrogels are created (Ju et al., 2023).

Compared with natural polymers, synthetic polymers contain particular molecular weights and fundamental structural units, and can be pre-designed to get desired qualities, such as particular porosity, degradation times, and tensile properties (Sánchez-Cid et al., 2022).

Synthetic hydrogels have a reliable material source and a longer quality guarantee period, with wider varieties and stable properties. However, the synthesis process may introduce chemical cross-linking agents and other toxic components. Their degradability and biocompatibility are poor (El-Sherbiny and Yacoub, 2013; Naahidi et al., 2017; Catoira et al., 2019).

### Hydrogels classification

**FIGURE 4:**
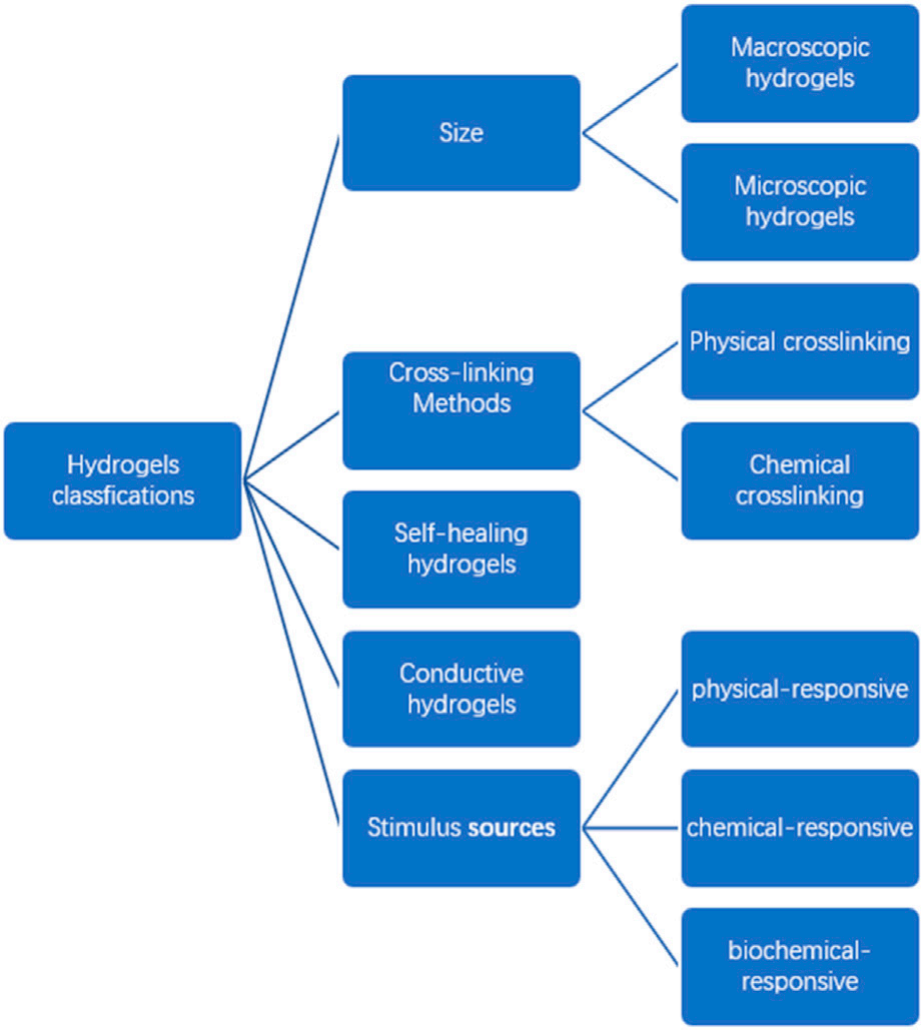
Hydrogels classification.

There are different types of hydrogels according to different classification methods. Except for the natural hydrogels and synthetic hydrogels mentioned above, we classify the hydrogels by their size, crosslinking modes, and properties (See Figure 4).

#### Size

According to the different sizes and shapes of hydrogels, there are macroscopic gels and microscopic gels (microspheres). Macroscopic gels can be divided into columnar, porous sponge, fibrous, membrane, and spherical according to the different shapes. The microscopic gels include micron-sized microspheres and nano-sized microspheres.

Particulate hydrogels with particle sizes in the micron and nanometer ranges, respectively, are known as microgels (hydrogel microspheres) and nanogels. Microgels and nanogels can be directly injected since they are significantly smaller than the inner diameter of a syringe needle than macroscopic hydrogels. Additionally, their greater relative surface area improves the capacity to penetrate tissue barriers and aids natural clearance. Different hydrogel sizes are suited for various drug delivery methods. For instance, microgels with a diameter of fewer than 5 nm are typically employed for pulmonary or oral delivery but are frequently viewed as unsuitable for intravascular injection due to their quicker circulation clearance rates. Because they can exit tiny blood arteries through the windows opened by the endothelium lining, nanogels with diameters between 10 and 100 nm are appropriate for systemic delivery and allow extravasation into tissue (Li and Mooney, 2016).

#### Cross-linking methods

Hydrogels can be divided into physical crosslinking and chemical crosslinking according to the bonding mode of the network structure.

Physical crosslinking refers to the formation of a cross-linked network structure through polymer chain entangling, crystallization points, or other weak interactions (such as ionic bonds, van der Waals forces, hydrogen bonds, etc.) without the formation of new bonds. Due to the formation of cluster structures between molecules during physical crosslinking, the uniformity of crosslinking is poor. Moreover, the mechanical strength and gel time of hydrogel were affected and the degradation process was hindered due to the defects of the network structure.

Chemical crosslinking refers to the formation of new covalent bonds, which form a three-dimensional network structure between molecules through copolymerization or condensation reaction. This kind of hydrogel is permanent and irreversible, and cannot be dissolved or fused by heating, which is also known as true gel. The common bonds and modes of chemical crosslinking are Schiff base reaction, Michael addition reaction, and light/heat initiated crosslinking.

#### Self-healing hydrogels

The use of self-healing hydrogels in 3D printing, medication delivery, and tissue regeneration has shown considerable promise (Mondal and Chatterjee, 2022; Bertsch et al., 2023). Self-healing hydrogels are often created using dynamic covalent bonding or non-covalent interaction principles. These hydrogels’ capacity for self-healing enables them to conform to damaged tissues and organs, hence enabling their protection. Additionally, the self-healing hydrogels have injectable qualities: under high shear conditions, it briefly become fluid before returning to their gel state. Importantly, the self-healing hydrogel is physically stable in place, making it possible for the encapsulated drug to be protected for a longer period of time and, as a result, to release drugs slowly. Physical crosslinking causes the shear-thinning characteristic (Ju et al., 2023).

#### Conductive hydrogels

Cellular actions that can encourage cytokine release and enhance the microenvironment of damaged tissue are controlled by bioelectrical signals, which are essential. In order to create composite conductive hydrogels, conductive nanomaterials like graphene and carbon nanotubes as well as conductive polymers like polyaniline and polypyrrole are frequently integrated into hydrogel networks (Walker et al., 2019).

#### Stimulus sources

Stimuli-responsive hydrogels are aqueous-swollen polymer networks that have the ability to perform a volume phase transition on the basis of external stimuli (Jalili et al., 2017). They can respond to a variety of external stimuli, changing their structure, physical makeup, chemical composition, or mechanical properties (Mellati et al., 2021).

Hydrogels are primarily split into three categories based on the types of stimulus sources: physical-responsive hydrogels, chemical-responsive hydrogels, and biochemical-responsive hydrogels. By the design of polymer molecules, the character of the hydrogels can be changed, which makes them more “smart” (Chang et al., 2022).

### Hydrogels and bone tissue repair

Bone tissue engineering, as a new technological innovation, helps to create three-dimensional substitutes similar to the human bone tissue thereby maintaining the bone’s structural as well as functional integrity. Scaffolds play a vital role in the bone tissue engineering aspect, which forms the bioengineered structure. Among the scaffold materials, hydrogels have porous structures which simulate the extracellular matrix and can serve as a carrier to facilitate growth factor promotion (Nallusamy and Das, 2021). With their hydrophilic nature, three-dimensional structure, and comparable ECM components, hydrogels are appropriate scaffolds for cellular infiltration, adhesion, growth, proliferation, migration, and differentiation. They are also easily chemically changeable and can be further modified to demonstrate a favorable degradation profile and mechanical integrity. It has been demonstrated that hydrogels derived from natural bone tissue, particularly those from periosteal and demineralized bone matrix sources, can promote osteoconduction and osteoinduction (Xia and Chen, 2022).

## Hydrogels coated exosomes

Although exosomes have many benefits and their therapeutic effect are promising, they still have some drawbacks (Shiue et al., 2019). Exosomes must be absorbed by the targeted cell by endocytosis in order for their biological effects to be triggered; otherwise, they would be quickly eliminated from the blood circulation and might even build up in the liver, spleen, lungs, and digestive system. Direct injections of exosomes intravenously, intraperitoneally, or subcutaneously may cause macrophages in the reticuloendothelial system to respond, which may result in their rejection. After interacting with sweat, tears, and the epithelial barrier (tight junctions), bodily and topical treatments on the skin or ocular surfaces have demonstrated limited half-lives. What’s more, the costly manufacturing methods that demand consistency and purity of nanometer-sized biomaterials are the root of the challenges in exosome purification and mass production. Exosome delivery, therefore, requires a more effective means of avoiding clearance by the host organism (Khayambashi et al., 2021). The most appropriate way to use exosomes in regenerative medicine is to combine them with biomaterials (Shi et al., 2017). Hydrogels, as versatile nanomaterials which can mimic natural tissue, have been extensively utilized as a vehicle for the local medication delivery of treated exosomes. Hydrogels’ hydrophilic and cross-linking properties aid in their capacity for controlled medication release (Khayambashi et al., 2021). Exosomes from various cell origins have so far been enclosed in hydrogels made of HA, gelatin, chitosan, and polypeptides (Qin et al., 2016; Liu et al., 2017).

### Preparation methods of hydrogels coated exosomes

**FIGURE 5:**
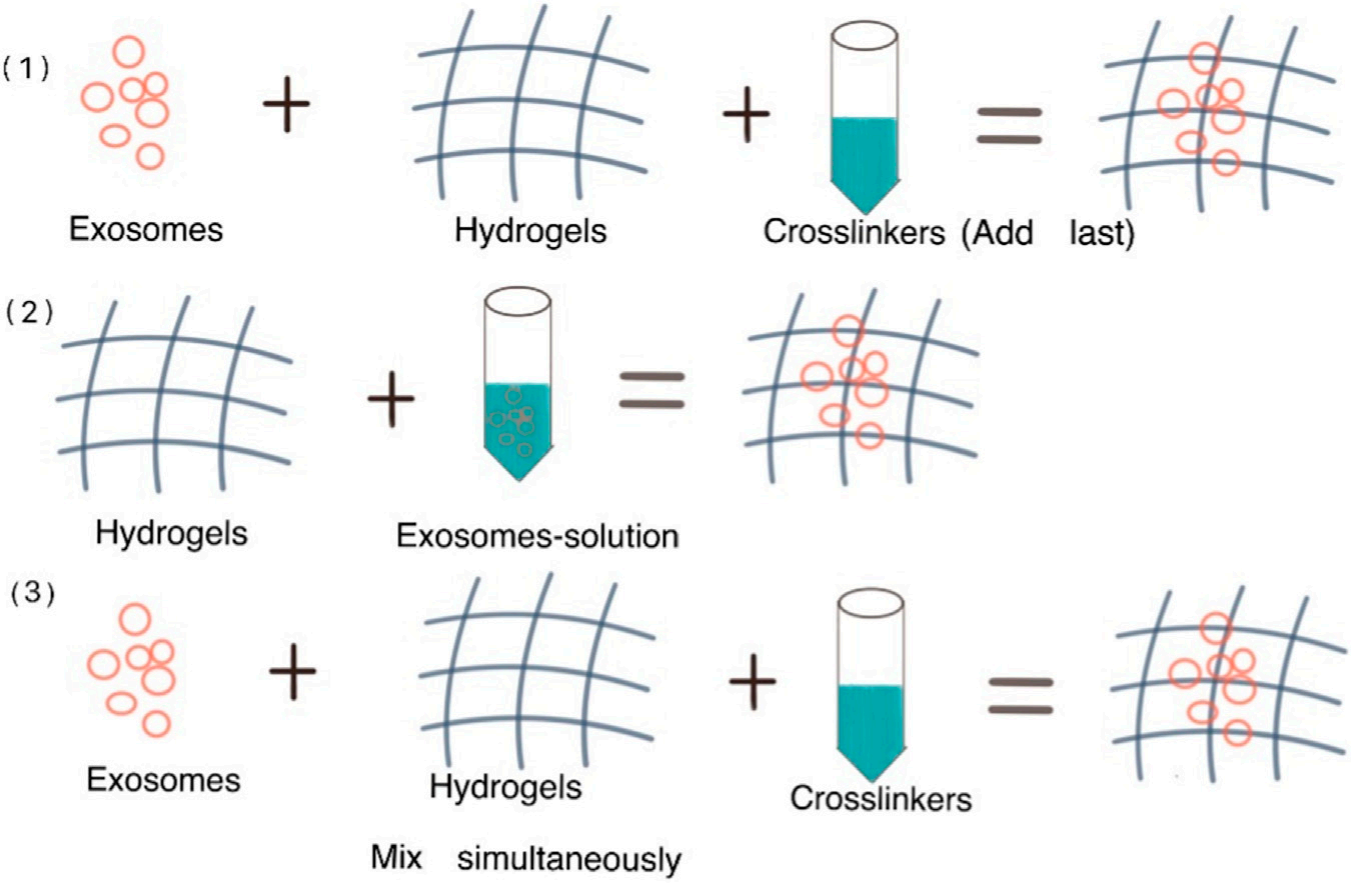
Methods to make hydrogels coated exosomes.

Three strategies were mentioned in a review by Parisa Khayambashi et al. to make hydrogels coated exosomes: 1) Exosomes and hydrogels are combined, then crosslinkers are added to cause gelation. 2) Physical incorporation of hydrogels or “breathing” technique. (Two basic steps are involved: the already inflated hydrogels are submerged in a solvent in order to remove the water from the hydrogel; then the hydrogels are soaked in an aqueous solution containing the exosomes, making the breathing-in of the exosomes into the porous hydrogel. 3) Simultaneous blending of the exosomes with the crosslinkers and the exosomes in solution, causing an *in situ* gelation that enables the exosomes to be delivered specifically (Khayambashi et al., 2021). Figure 5 demonstrates the preparation methods of hydrogels coated exosomes.

### Hydrogels coated exosomes and bone diseases

**FIGURE 6:**
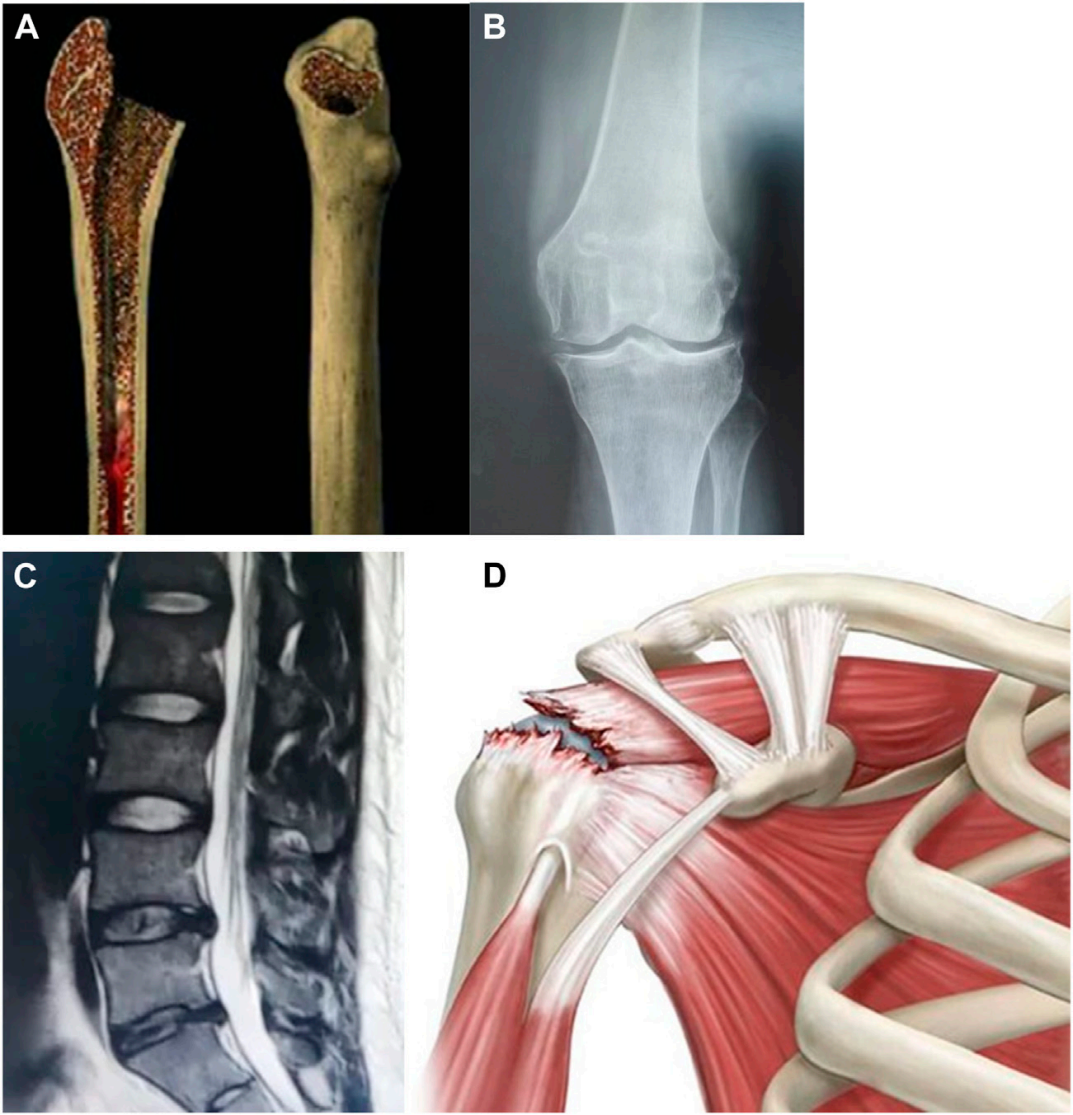
Hydrogels-coated exosomes have a potential therapeutic effect on bone diseases. **(A)**: Bone defect **(B)**: Knee osteoarthritis **(C)**: Intervertebral disc degeneration **(D)**: Rotator cuff tear.

We have summarized the application of hydrogels coated exosomes in bone defect, osteoarthritis/cartilage defect, intervertebral disc degeneration, and rotator cuff tear/tendon repair (See Figure 6). Previous studies showed the potential therapeutic effect of hydrogels coated exosomes on these diseases.

#### Hydrogels coated exosomes in bone defect

A safe and effective treatment must be utilized to achieve bone tissue regeneration and repair since bone defects brought on by trauma, bone tumor removal, infection (such as osteomyelitis) and other diseases have emerged as major problems substantially impacting patients’ limb function (Chang et al., 2022). There are studies showing hydrogels coated exosomes have the potential therapeutic effect on the bone defect. Li Wang et al. studied a new type of self-healing hydrogels using coralline hydroxyapatite (CHA)/silk fibroin (SF)/glycol chitosan (GCS)/difunctionalized polyethylene glycol (DF-PEG) and made it a carrier of HucMSCs (human umbilical cord) derived exosomes. They injected the mixture of exosomes and hydrogels into the femoral condyle defect area in SD rats and found the bone defect area had effectively healed (Wang et al., 2020b). In another study, Zhang Yuntong et al. used hyaluronic acid hydrogel (HA-Gel)-encapsulated umbilical MSC-derived exosomes (uMSCEXOs) in conjunction with specialized nanohydroxyapatite/polycaprolactone (nHP) scaffold folds to heal cranial lesions in rats and demonstrated the uMSCEXOs combined with the novel composite can stimulate both angiogenesis and osteogenesis in a critical-size cranial defect model (Zhang et al., 2021a). Rui Li et al. injected the adipose-derived exosomes loaded by gelatine hydrogels into a rat skull defect model and found it can regulate bone immune metabolism and promote bone healing (Li et al., 2022).

#### Hydrogels coated exosomes in osteoarthritis/cartilage defect

Osteoarthritis (OA) is a common bone disease that can cause joint pain, stiffness, and limited activity, which is the main cause of disability in the elderly. OA is primarily characterized by degenerative cartilage lesions. Chondrocytes and extracellular matrix make up the majority of the cartilage in the joint while there are no blood vessels or nerves. Chondrocytes, which are highly differentiated cells, react primarily by synthesizing and secreting the cartilage matrix, which is crucial for preserving the metabolic equilibrium of cartilage tissue (Liang et al., 2021b). There is currently no medication that can stop the progression of OA. Surgical intervention may solve the problems, but any surgery has risks and the possibility of failure. To date, injecting therapeutic materials into the joint cavity has become a new treatment method for osteoarthritis. And hydrogels-coated exosomes have been reported to have the function of attenuating cartilage degradation and enhancing cartilage regeneration, which shows an attractive prospect in the treatment of osteoarthritis (Zhang et al., 2021c; Luo et al., 2022; Shen et al., 2022). Yu Zhang et al. studied the thermosensitive gel with poloxamers as a delivery platform for PRP (platelet-rich plasma) -Exosomes, finding the new therapy can induce cartilage proliferation and inhibit cartilage degradation in subtalar osteoarthritis rat model (Zhang et al., 2022). Xuehan Sang et al. used thermosensitive hydrogel loaded with chondrocyte-derived exosomes to treat damaged cartilage and proved its function to relieve OA through positively regulating chondrocytes on the proliferation, migration, and differentiation (Sang et al., 2022). Gelatin methacrylate loaded with MSCs exosomes was described to fabricate a 3D-printed decellularized extracellular matrix (ECM), according to Chen et al. (Mahmood et al., 2022). This technology made it possible to create radically directed channels, which improve cartilage regeneration by controlling chondrocyte migration and healing osteochondral defects. Fang-Xue Zhang et al. (Zhang et al., 2021c) discussed the effect of an injectable mussel-inspired highly adhesive hydrogel with exosomes to repair cartilage defects in rat patellar grooves, which can be a potential treatment for cartilage defects. Xiaolin Liu et al. reported photoinduced imine crosslinking (PIC) hydrogel coated MSCs exosomes and demonstrated its ability to facilitate articular cartilage regeneration (Liu et al., 2017).

#### Hydrogels coated exosomes in intervertebral disc degeneration

The intervertebral disc has the function of increasing the range of spinal motion, bearing pressure, cushioning vibration, and protecting the brain and spinal cord. Similar to osteoarthritis, degenerative intervertebral disc disease is also a common orthopedic condition, having a severe impact on people’s life quality. Intervertebral disc consists of fibrous ring, nucleus pulposus and hyaline cartilage plate. The ECM’s imbalance of catabolism and anabolism as well as changes in the intervertebral disc microenvironment are the primary causes of intervertebral disc degeneration (Binch et al., 2021). MSCs transplantation can be a treatment method for intervertebral disc degeneration (Sakai and Andersson, 2015). However, stem cell transplantation still faces potential risks of *in vivo* survival, immunogenicity, and tumorigenicity (Hao et al., 2017). MSCs-derived exosomes can inherit MSCs’ properties, restrain the aptosis of nucleus pulposus cells and promote ECM synthesis, thus mitigating inflammatory responses. What’s more, low immunogenicity makes exosomes an ideal therapeutic material for intervertebral disc degeneration (Xing et al., 2021). However, the interaction time of exosome injection alone is too short to achieve long-term effects. So, it’s proper to load the exosomes into a container. Hydrogels, as mentioned before, show stable property and biocompatibility and can serve as the carrier of exosomes. As for the applications of hydrogels coated exosomes on intervertebral disc degeneration, some studies have been done. Liwen Luo et al. tried to inhibit intervertebral disc degeneration by injecting transcostal cartilage ECM modified hydrogels coated exosomes from cartilage endplate stem cells into the impaired intervertebral disc. They collected intervertebral disc samples from rats and patients who underwent elective intervertebral removal surgery. The outcomes revealed that the compound material can release exosomes stably and prevent the degeneration of intervertebral disc (Luo et al., 2022). Ming Guan et al. developed an injectable, self-healing biocompatible hydrogel to load MSCs-Exosomes and drew the conclusion that the hydrogels coated exosomes can treat disc degeneration by attenuating nucleus pulposus cellular senescence (Guan et al., 2023).

#### Hydrogels coated exosomes in rotator cuff tear/tendon repair

The rotator cuff is composed of the tendons of the supraspinatus, infraspinatus, teres minor and subscapularis muscles. It’s an important structure to maintain shoulder stability. Rotator cuff tear is a common injury in sports. Tendon-bone interface recovery is the key factor in rotator cuff tear repair. The formation of fibrovascular scar tissue after rotator cuff injury can affect tendon-bone healing (Gulotta et al., 2009). Currently, biological intervention is applied to treat rotator cuff tear to promote tendon-bone healing, which may be crucial for preventing retear (Zhang et al., 2021d). With the discovery of exosomes and the emergence of various hydrogels, there are researches focusing on rotator cuff tear repair using the combination of the two materials. Jiangyu Cai et al. evaluated the effect of sodium alginate hydrogel coated Kartogenin-Preconditioned MSCs exosomes on the rotator cuff tear model of the rat. The outcomes showed that it can accelerate the rotator cuff tendon-bone repair, enhancing its biomechanical properties (Cai et al., 2023). Xiaopeng Tong et al. analyzed the function of hydrogels coated human urine-derived stem cells (USCs) exosomes on a rat rotator cuff tear model. The results proved its ability to heal tendon-bone interface (Tong et al., 2023). Jinwei Lu et al. developed GelMA hydrogels coated platelet-derived exosomes and verified their capacity to promote tendon stem/progenitor cell proliferation and regeneration, facilitating tendon repair (Lu et al., 2023).

## Conclusion

Exosomes are a subset of extracellular vesicles with a diameter of 30–200 nm. Their applications in bone tissue repair have been studied. Hydrogels have 3D structure work and can be used in tissue repair as well as drug carriers. A combination of the exosomes and the hydrogels may utilize their advantages and make up for the disadvantages. We summarize the hydrogels coated exosomes in bone tissue repair and reviewed their therapeutic effects on the bone defect, osteoarthritis/cartilage defect, intervertebral disc degeneration, and rotator cuff tear/tendon repair. Hydrogels coated exosomes are promising, and their role in bone tissue repair needs to be further explored. In the future, through the improvement of exosomes extraction and hydrogels synthesis, hydrogels coated exosomes are expected to play a greater role in the diagnosis and treatment of orthopedic diseases, solving bone tissue repair problems.
